# Treatment Patterns of Diabetes in Italy: A Population-Based Study

**DOI:** 10.3389/fphar.2019.00870

**Published:** 2019-08-06

**Authors:** Aida Moreno Juste, Enrica Menditto, Valentina Orlando, Valeria Marina Monetti, Antonio Gimeno Miguel, Francisca González Rubio, María Mercedes Aza–Pascual-Salcedo, Caitriona Cahir, Alexandra Prados Torres, Gabriele Riccardi

**Affiliations:** ^1^EpiChron Research Group, Aragon Health Sciences Institute (IACS), IIS Aragon, REDISSEC ISCIII. Miguel Servet University Hospital, Zaragoza, Spain; ^2^Aragon Health Service (SALUD), Department III of Zaragoza, Zaragoza, Spain; ^3^CIRFF, Center of Pharmacoeconomics, University of Naples Federico II, Naples, Italy; ^4^Division of Population Health Sciences, Royal College of Surgeons in Ireland, Dublin, Ireland; ^5^Diabetes, Nutrition and Metabolism research group, Department of clinical Medicine and Surgery, Frederico II Universityn, Naples, Italy

**Keywords:** antidiabetic drugs, pattern, treatment switching, treatment addition, persistence

## Abstract

**Background:** The steady increase in type 2 diabetes prevalence and the availability of new antidiabetic drugs (AD) have risen the use of these drugs with a change in the patterns of specific drug utilization. The complexity of this treatment is due to successive treatment initiation, switching and addition in order to maintain glycaemic control. The aim of this study was to describe the utilization patterns of ADs at initiation, treatment addition, and switching profiles and to measure factors influencing persistence to therapy.

**Methods:** Retrospective observational study. Data were retrieved from the Campania Regional Database for Medication Consumption. Population consisted of patients receiving at least one prescription of ADs between January 1 and December 31, 2016. We calculated time to treatment switching or add-on as median number of days and interquartile range (IQR). Persistence rates were estimated using the Kaplan–Meier method. We used Cox regression models to estimate the likelihood of non-persistence over 1 year of follow-up. Hazard ratios and 95% confidence intervals were calculated.

**Results:** Of 14,679 patients, 86.9% started with monotherapy and 13.1% with combination therapy. Most common initial treatment was metformin in both monotherapy and combination therapy. First-line prescription of sulfonylurea was observed in 6.9% of patients aged 60–79 years and in 10.8% of patients aged ≥80 years. Patients starting with metformin showed fewer treatment modifications (10.4%) compared to patients initiating with sulfonylureas (35.2%). Newer ADs were utilized during treatment progression. Patients who initiated with sulfonylurea were approximately 70% more likely to discontinue treatment compared to those initiated on metformin. Oldest age group (≥80 years) was more likely to be non-persistent, and likelihood of non-persistence was highest in polymedicated patients. Patients changing therapy were more likely to be persistent.

**Conclusions:** Our results show that treatment of T2D in Italy is consistent with clinical guidelines. Even if newer ADs were utilized during disease progression, they seem not to be preferred in patients with a higher comorbidity score, although these patients could benefit from this kind of treatment. Our study highlights patients’ characteristics that might help identify those who would benefit from counselling from their health-care practitioner on better AD usage.

## Introduction

Type 2 diabetes (T2D) is widely considered one of the world’s largest human health problems, as documented by its growing prevalence in recent decades (Baviera et al., 2011). In 2016, it was reported that more than 3.2 million people in Italy suffer from diabetes, 5.3% of the total population (Gargiulo et al., 2017). Over the last decade, several new antidiabetic drugs (ADs), with varying clinical efficacy, profiles, and costs, are being introduced in the market, enabling physicians to tailor therapy for each individual patient (Grimes et al., 2015; Orlando et al., 2015). The steady increase in T2D prevalence and the availability of these new medicines have resulted in increased AD utilization and related costs worldwide, with a number of studies showing a change in specific drug utilization patterns and an increase in prescribing for T2D over time (Grimes et al., 2015; Rafaniello et al., 2015). In addition, the treatment of T2D of each patient changes by successive initiating, adding, and switching of drugs with different mechanisms in order to maintain glycemic control (Lamberts et al., 2011), and these factors increase the complexity of the treatment. Therefore, suboptimal glycemic control can be influenced by the healthcare practitioner whether it be a general practitioner (GP) or diabetologist (Khunti et al., 2018). In Italy, reimbursement legislation does not allow GPs to prescribe autonomously new ADs, such as SGLT2 inhibitors or GLP-1Ras, without an official specialist’s approval. This could influence the choice of treatment at initiation and the trend of drug utilization observed. Another key factor for long-term success of pharmacotherapy in T2D is the dependence on patients continuing to take their medications as prescribed (O’Shea et al., 2015). The term “medication persistence” refers to the act of conforming to a recommendation of continuing treatment for the prescribed length of time (Vrijens et al., 2012). Early discontinuation of the prescribed treatment is defined “non-persistence.” Suboptimal persistence can lead to compromised health outcomes (e.g., higher risk of hospitalizations and emergency room visits, increased morbidity, and premature mortality) and wasted time and money with serious economic consequences (Rascati et al., 2017). This is highly relevant in the treatment of T2D given that this condition is chronic and typically requires long-term commitment to therapeutic regimens to gain and maintain glycemic control and, consequently, prevent complications (Gregoire et al., 2010).

On the other hand, comorbidity is present in most patients with T2D, and studies have suggested that increased number and severity of comorbid diseases may, in turn, affect persistence and adherence to antidiabetic medication (O’Shea and Teeling, 2013; O’Shea et al., 2015; Simard et al., 2015).

In light of the recent introduction on the market of new antidiabetics, the aim of this study was i) to describe the utilization patterns of ADs at initiation, ii) to describe treatment addition and switching (i.e., regimen change) profiles, and iii) to measure persistence and investigate factors related to non-persistence.

## Methodology

### Study Design and Population

It was conducted a retrospective observational non-interventional administrative database study in the primary care setting of Campania, one of the largest Italian regions situated in the south of the country representing about 10% (i.e., 5.9 million inhabitants) of the Italian population. As in all other Italian regions, health care services (free or at a nominal charge) are provided to all citizens and legal foreign residents through Local Health Units (LHUs). About 99% of them are covered by the public healthcare system.

The source population consisted of people living in the area of four LHUs, representing about 60% of the total Campania population. All the patients that had these characteristics were included in the study: i) patients aged 40 years and older; ii) patients who had received at least one prescription of antidiabetic drugs between January 1 and December 31, 2016; iii) patients who were alive and registered in the list of LHUs for at least 2 years before and after the index date (i.e., the date of first prescription of an AD); and iv) patients without any recorded AD prescription in the two years preceding the index date. Patients receiving only one prescription (spot users) are excluded from the analysis.

### Data Source

Data necessary for the study were retrieved from the Campania Regional Database for Medication Consumption containing records of drugs dispensed by community pharmacies and reimbursed by Local Health Authorities (LHUs). This database provides the following information for each prescription: anonymous patient code, date of dispensation, Anatomical Therapeutic Chemical (ATC) code, number of Defined Daily Doses (DDD), number of packages dispensed, and drug price. The database is matched, by record-linkage analysis, to the civil registry to collect demographic information. This database has been used previously in drug-utilization studies (Iolascon et al., 2013; Casula et al., 2014; Iolascon et al., 2016; Putignano et al., 2017). Data sources were matched by record linkage analysis through a unique and anonymous personal identification code. Such code was created by a database manager, uninvolved in the data analysis, preventing patient identification. Permission use anonymized data to this study was granted to the researchers of the Centro di Ricerca in Farmacoeconomia e Farmacoutilizzazione (CIRFF) by the governance board of Unità del Farmaco della Regione Campania. The CIRFF has a regional decree that allow for conducting research by making secondary use of administrative data (DGRC n 276 23/05/2017). The article does not contain clinical studies, and all patients’ data were fully anonymized and were analysed retrospectively. For this type of study, formal consent is not required according to current national law from Italian Medicines Agency.

### Patterns of Utilization of ADs and Treatment Switching and Addition

New users of ADs were stratified in different categories according to their first prescription during the study period: metformin, sulfonylureas, alpha-glucosidase inhibitors, dipeptidyl peptidase-4 (DPP-4) inhibitors, repaglinide, other monotherapy including thiazolidinediones, glucagon-like peptide-1 receptor agonists (GLP-1Ras), and sodium-glucose co-transporter-2 (SGLT2) inhibitors. Patients to whom were prescribed combinations of oral blood glucose-lowering drugs were classified as fixed-combination therapy. Patients receiving prescription of two different ADs with an overlapping period of at least 15 days were classified as free-combination therapy, accordingly with previous studies (Overbeek et al., 2017).

The consistency of the treatment patterns, identified by the analysis, with clinical guidelines was independently assessed by three clinicians from the research team with proven expertise in the field of diabetes care (AMJ, FGB, and GR).

For each patient, it was assessed the following variables at baseline: age, sex, number of concomitant drugs (polypharmacy), area of living, use of neuro-psychiatric drugs, macro- and microvascular complications (Cammarota et al., 2014), and comorbidity score. The patients were stratified into three age groups: 40–59, 60–79, and ≥80 years. The number of concomitant drug was classified in three groups: 0–5 (no polypharmacy), 6–9 (polypharmacy), and ≥10 drugs (excessive polypharmacy). The comorbidity score was evaluated using the RxRisk index. It is a validated measure for determining an individual’s comorbidity based on their medicine dispensing. It was developed using therapeutic drug classes and medicinal agents for selected chronic comorbidities (Pratt et al., 2018). The list of comorbidities and drugs used in the score is summarized in **Supplementary Table 1** in the supporting information (Pratt et al., 2018), excluding diabetes from the list as it was the index disease.

The utilization patterns of ADs were analyzed within 365 days from treatment initiation. Treatment switching was defined as discontinuation of initial antidiabetic drug and initiation on an alternative agent from a different drug class. Patients switching back to their initial therapy within 30 days were not classified as switchers. Add-on therapy was considered as receiving prescription of a different therapeutic class while continuing their first treatment. In the add-on therapy evaluation, fixed-combination was considered add-on of each single active agent. It was also evaluated dose change (increasing or decreasing dosage) of the initial medication within therapeutic class.

### Measuring Persistence

Persistence was defined as continuation of treatment during 1 year from the index date, and it was estimated by measuring the time gap between a drug dispensation and the following one. Patients were considered non-persistent if the gap between two refills was over two and a half times the duration of the preceding prescription (grace period), based on sensitivity analyses from previous research (Malo et al., 2017; Menditto et al., 2018). The number of days of medication supplied was estimated based on the number of pills and packages. Medication persistence was measured at the drug class level. It was not considered as an interruption the switching products within index medication classes. Patients were censored if the gap allowed was exceeded without purchasing a new prescription or upon reaching the end of the study period (if they had been persistent throughout the follow-up period). Non-persistent users were categorized as users who restarted AD therapy after a period of discontinuation or users who simply discontinued treatment.

### Statistical Analysis

A descriptive analysis of patient characteristics and initial treatment patterns was performed. The time to treatment switching or add-on was calculated as median number of days and interquartile range (IQR). Therefore, differences between patient characteristics were compared using chi-square test for categorical variables or unpaired t test for numerical variables, as appropriate.

Persistence rates were estimated using the Kaplan–Meier method. Statistical differences between curves were assessed using the log-rank test, and Cox regression models estimated the likelihood of non-persistence over 1 year after AD initiation and evaluated the factors affecting persistence. Hazard ratios (HRs) and 95% confidence intervals (95%CI) were calculated assessing crude and adjusted associations for relevant predictors.

Data management was performed with Microsoft SQL server (version 2018), and all analyses were performed with SPSS software for Windows (version 17.1, SPSS Inc., Chicago, IL, USA). *P* value <0.05 was considered significant.

## Results

### Overall Study Population Characteristics

**Figure 1:**
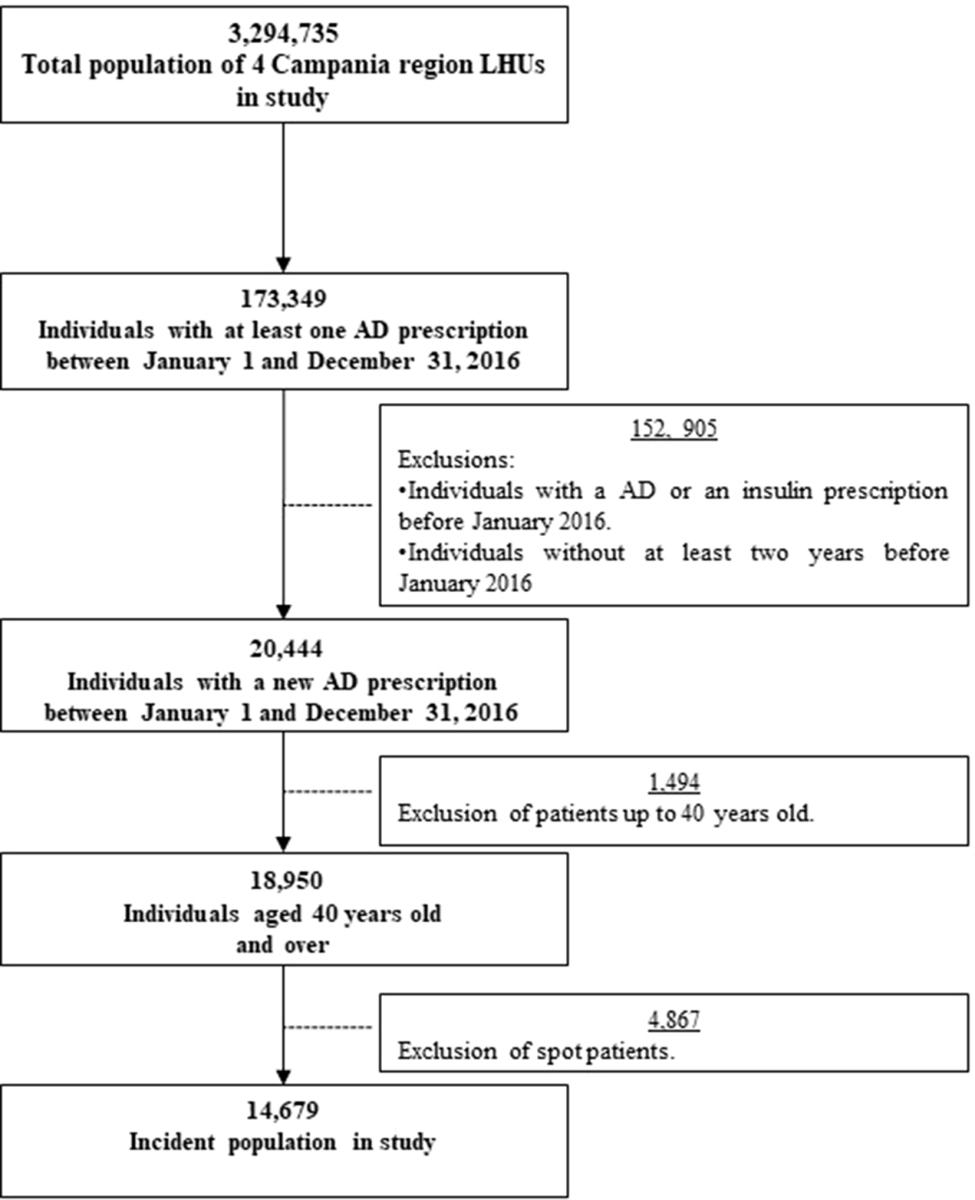
Flow chart of the study.

A total of 19,546 patients aged over 40 years were new users of antidiabetic drugs. Of these, 4,867 (25.68%) were spot users, defined as patients receiving only one prescription of the drug. Most of them were between 60 and 79 years old. The spot users had a monotherapy prescription in 78.6% of the patients and 67.6% of them had a metformin prescription, followed by sulfonylureas (**Supplementary Table 2**). A total of 14,679 patients were included in the study (**Figure 1**). A significantly larger proportion of males were present in the cohort (54.8% *vs*. 45.2%, *P* < 0.001), and the majority of patients were living in an urban area (*N* = 91.2%). The mean age (± SD) of the cohort was 64 ± 11.6 years. Over the 2 years prior to index date (cohort entry), 554 patients (3.8%) had microvascular or macrovascular complications. About 18.7% of the cohort at entry used drugs for mental health disorders. It was observed that 41.2% of the new users of AD were prescribed up to 5 comedications, 24.1% between 6 and 9, and 34.7% over 10. The average comorbidity score, calculated as mean number of chronic comorbidities per the RxRisk index, was 3.3 ± 2.7 (**Table 1**).

**Table 1:** Demographic and clinical characteristics of new users of antidiabetics at cohort entry.

Characteristics	Monotherapy *N* = 12,753 (86.9%)						Combination therapy N = 1,926 (13.1%)		Total *N* = 14,679 (100%)	*P*-value
	Metformin *N* = 10,246	Sulfonylureas *N* = 982	Alpha glucosidase inhibitors *N* = 638	^£^ DPP-4 *N* = 161	Repaglinide *N* = 583	°Other monotherapy *N* = 143	Fixed combination *N* = 1,426	Free combination *N* = 500		
**Age (MD** ± **SD)**	63.2 ± 11.3	66.8 ± 12.1	66.5 ± 11.8	68.1 ± 12.1	69.5 ± 12.4	59.0 ± 10	64.8 ± 12.2	62.8 ± 11.3	64.0 ± 11.6	<0.001*
** 40–59**	4,019 (74.3%)	283 (5.2%)	180 (3.3%)	37 (0.7%).	131 (2.4%)	75 (1.4%)	482 (8.9%)	202 (3.7%)	5,409 (100%)	
** 60–79**	5,380 (69.2%)	537 (6.9%)	361 (4.6%)	92 (1.2%)	316 (4.1%)	67 (0.9%)	762 (9.8%)	257 (3.3%)	7,772 (100%)	
** ≥80**	847 (56.5%)	162 (10.8%)	97 (6.5%)	32 (2.1%)	136 (9.1%)	1 (0.1%)	182 (12.1%)	41 (2.7%)	1,498 (100%)	
**Sex**										0.001*
** F**	4,639 (70.1%)	477 (7.2%)	304 (4.6%)	73 (1.1%)	275 (4.2%)	68 (1.0%)	591 (8.9%)	190 (2.9%)	6,617 (100%)	
** M**	5,590 (69.6%)	504 (6.3%)	333 (4.1%)	86 (1.1%)	307 (3.8%)	74 (0.9%)	830 (10.3%)	305 (3.8%)	8,029 (100%)	
**Polypharmacy**										<0.001*
** 0–5 (no-polypharmacy)**	4,106 (67.9%)	412 (6.8%)	204 (3.4%)	63 (1.0%)	183 (3.0%)	44 (0.7%)	752 (12.4%)	285 (4.7%)	6,049 (100%)	
** 6–9 (polypharmacy)**	2,567 (72.5%)	218 (6.2%)	146 (4.1%)	33 (0.9%)	129 (3.6%)	46 (1.3%)	304 (8.6%)	97 (2.7%)	3,540 (100%)	
** ≥10 (excessive polypharmacy)**	3,573 (70.2%)	352 (6.9%)	288 (5.7%)	65 (1.3%)	271 (5.3%)	53 (1.0%)	370 (7.3%)	118 (2.3%)	5,090 (100%)	
**Area of living**										0.047*
** Rural**	910 (71.7%)	93 (7.3%)	56 (4.4%)	9 (0.7%)	42 (3.3%)	20 (1.6%)	107 (8.4%)	33 (2.6%)	1,270 (100%)	
** Urban**	9,155 (69.7%)	873 (6.6%)	573 (4.4%)	150 (1.1%)	532 (4.1%)	122 (0.9%)	1,272 (9.7%)	451 (3.4%)	13,128 (100%)	
**Neuro-psychiatric drugs**										0.019*
** No**	8,335 (69.8%)	786 (6.6%)	495 (4.1%)	125 (1.0%)	469 (3.9%)	118 (1.0%)	1,177 (9.9%)	429 (3.6%)	11,934 (100%)	
** Yes**	1,911 (69.6%)	196 (7.1%)	143 (5.2%)	36 (1.3%)	114 (4.2%)	25 (0.9%)	249 (9.1%)	71 (2.6%)	2,745 (100%)	
**Micro/macrovascular complication**										<0.001*
** No**	9,899 (70.1%)	949 (6.7%)	613 (4.3%)	152 (1.1%)	527 (3.7%)	139 (1.0%)	1,364 (9.7%)	482 (3.4%)	14,125 (100%)	
** Yes**	347 (62.6%)	33 (6.0%)	25 (4.5%)	9 (1.6%)	56 (10.1%)	4 (0.7%)	62 (11.2%)	18 (3.2%)	554 (100%)	
**Comorbidity score**	3.3 ± 2.6	3.3 ± 2.8	3.9 ± 2.8	3.4 ± 2.5	4.1 ± 3.1	3.0 ± 2.6	2.8 ± 2.8	2.5 ± 2.6	3.3 ± 2.7	<0.001*

*P-value less of 0.05 was statistically significant. ^£^ DPP-4, dipeptidyl peptidase-4 inhibitors; °Other monotherapy, A10BG Thiazolidinediones (n = 21); A10BJ Glucagon-like peptide-1 analogue (n = 88); A10BK Sodium-glucose co-transporter 2 (SGLT2) inhibitors (n = 34).

### Patterns of Therapy Utilization

Of the 14,679 total patients, 86.9% were initiated with monotherapy and 13.1% with combination therapy. Among monotherapy, metformin was the most commonly prescribed (80.3% *n* = 10,246), followed by sulfonylureas (7.7% *n* = 982); 5% were initiated with alpha glucosidase inhibitors, 4.6% with repaglinide, and 1.3% with dipeptidyl peptidase-4 (DPP-4) inhibitor, and 1.1% of patients were prescribed a different antidiabetic agent in the index date (**Table 1**).

The most prescribed fixed combination was metformin and sulfonylureas (53% of the patients) followed by metformin and sitagliptin (12.6%) (**Supplementary Table 3**). About 50% of patients initiating on free combination therapy used metformin and sulfonylureas, followed by combination of metformin and repaglinide in 15% of cases.

Patients who initiated with repaglinide had a significant higher percentage of micro/macrovascular complications (10.1%), a significant higher comorbidity score (4.1 ± 3.1), and a significant higher percentage of patients aged more than 80 years (9.1%) compared to other initiation therapies. The characteristics of the population, stratified by type of therapy at cohort entry, are described in **Table 1**.

### Treatment Switching and Addition

Of those initiated on metformin, 10.4% had an episode of treatment switching (**Table 2**). The most frequent switches were to sulfonylureas (31.1%), repaglinide (16.5%), or SGLT-2 inhibitors (11.5%). The median time to treatment switching when initiated on metformin was 95 days (IQR 190). Patients in metformin treatment switched to insulin in 11.4% of the cases, with a median time of 150 days (IQR 210). Patients who switched from metformin to alpha-glucosidase or to repaglinide had a significant higher co-morbidity score (4.3 ± 2.8 and 4.1 ± 3.2, respectively). Of the sulfonylurea cohort, 35.2% of patients switched treatment with a median time of 44.5 days (IQR 136). The majority switched to metformin (73.1%), and 15.3% switched to DPP-4 inhibitors. Overall, 17% of patients switching therapy received a within-class change (dose change) prior to switching therapy. Most of these dose changes were from patients initiated on metformin. In the within treatment class changes for the metformin and sulfonylurea groups, dose increases were more frequent (69%) than decreases (31%). Among patients who initiated on metformin, 9% received treatment addition and 10.1% of patients initiated on a sulfonylurea (**Table 3**). For the metformin group, the most frequent additions were insulin (33.7%) followed by sulfonylurea (26.6%), DPP-4 inhibitors (20.7%), and SLGT-2 (10.1%). For those starting with sulfonylurea, the most frequent addition was metformin (66.7%), followed by insulin (31.3%). The median time to add-on therapy was shorter in the metformin group (51.2 days, IQR 132) than in the sulfonylurea group (90 days, IQR 182). About 25% of patients received a dose change of their initial medication prior to treatment add-on. Most of them were patients initiated on metformin (95%).

**Table 2:** Switching patterns among new users of antidiabetic drugs initiated on either metformin or a sulphonylurea.

Therapy at the index date	Total switchers	Treatment switching									
		Metformin	Sulfonylureas	Alpha glucosidase inhibitors	£DPP-4 inhibitor	Repaglinide	Thiazolidinediones	GLP-1	1SGLT2	Insulin	*P*-value
**Total N = 11,228**	1,414 (12.6%)	253 (17.9%)	340 (24.1%)	109 (7.7%)	158 (11.2%)	197 (13.9%)	33 (2.3%)	108 (7.6%)	133 (9.4%)	147 (10.4%)	
**Metformin: (** ***N*** ** = 10,246)**	1,068 (10.4%)	–	332 (31.1%)	98 (9.2%)	105 (9.8%)	176 (16.5%)	22 (2.1%)	102 (9.6%)	123 (11.5%)	122 (11.4%)	<0.001*
** Comorbidity score (Mean ± SD)**	3.5 ± 2.8		3.4 ± 2.9	4.3 ± 2.8	3.2 ± 2.6	4.1 ± 3.2	3.5 ± 3.0	2.5 ± 2.1	2.6 ± 2.1	3.9 ± 3.0	<0.001*

** Sulfonylurea: (** ***N*** ** = 982)**	346 (35.2%)	253 (73.1%)	8 (2.3%)	11 (3.2%)	53 (15.3%)	21 (6.1%)	11 (2.9%)	6 (1.7%)	10 (2.9%)	25 (7.2%)	0.610
**Comorbidity score (Mean ± SD)**	3.2 ± 3.0	3.1 ± 2.7	5.8 ± 7.2	3.2 ± 2.6	2.6 ± 3.0	4.8 ± 3.0	3.7 ± 2.8	2.8 ± 3.0	2.6 ± 2.2	2.6 ± 2.8	0.125

*P-value less of 0.05 was considered to be statistically significant. £ DPP-4, dipeptidyl peptidase-4 inhibitors; ºGLP-1, glucagon-like peptide-1 receptor agonists; 1 SGLT-2, sodium-glucose co-transporter-2 inhibitors.

**Table 3:** Treatment add-on patterns among new users of antidiabetic drugs initiated on either metformin or a sulphonylurea.

Therapy at the index date	Total add-on	Treatment add-on									
		Metformin	Sulfonylureas	Alpha glucosidase inhibitors	^£^DPP-4 inhibitor	Repaglinide	Thiazolidinediones	ºGLP-1	^1^SGLT2	Insulin	*P-value
**Total** ***N*** ** = 11,228**	1,017 (9.1%)	66 (6.5%)	244 (24.0%)	14 (1.4%)	192 (18.9%)	39 (3.8%)	13 (1.3%)	16 (1.6%)	93 (9.1%)	340 (33.4%)	–
**Metformin: (** ***N*** ** = 10,246)**	918 (9.0%)	–	244 (26.6%)	14 (1.5%)	190 (20.7%)	39 (4.2%)	13 (1.4%)	16 (1.7%)	93 (10.1%)	309 (33.7%)	0.432
** Comorbidity score (Mean ± SD)**	2.8 ± 2.5		3.2 ± 2.8	3.6 ± 3.5	2.8 ± 2.5	3.7 ± 2.9	3.5 ± 2.4	2.6 ± 2.4	2.8 ± 2.2	2.6 ± 2.5	0.407
**Sulfonylureas: (** ***N*** ** = 982)**	99 (10.1%**)**	66 (66.7%)	–	–	2 (2.0%)	–	–	–	–	31 (31.3%)	0.067
** Comorbidity score (Mean ± SD)**	2.4 ± 2.5	2.8 ± 2.7	–	–	3.0 ± 0.0	–	–	–	–	2.2 ± 2.3	0.143

*P-value less of 0.05 was considered to be statistically significant. ^£^ DPP-4, dipeptidyl peptidase-4 inhibitors; ºGLP-1, glucagon-like peptide-1 receptor agonists; 1 SGLT-2, sodium-glucose co-transporter-2 inhibitors.

### Persistence

**Figure 2:**
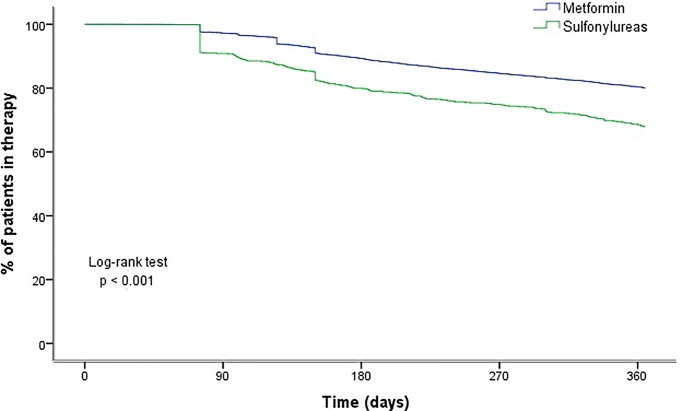
Persistence with antidiabetic drugs at 1 year after initiation, by drug class.

In the analysis of persistence, 11,228 patients were included. Overall, 79% of the patients were still taking their therapy after 12 months of treatment initiation. Persistence varied depending on the antidiabetic agent; while 80.1% of patients on metformin persisted with their therapy 12 months after initiation, only 67.9% of those on sulfonylurea were persistent. For those on metformin, the average period between the index date and treatment discontinuation was 330 days (95%CI 328.6; 331.7), while it was 303 days (95%CI 296.6; 309.7) for those on sulfonylurea. According to Kaplan–Meier analysis, differences in persistence rates were observed according to the type of treatment at initiation (log-rank, *P* < 0.0001) (**Figure 2**).

Cox regression analysis (**Table 4**) showed that the oldest age group (≥80 years) was more likely to be non-persistent than the younger age group. Patients who initiated with a sulfonylurea were approximately 70% more likely to have a period of discontinuation compared to those initiated on metformin. Patients living in an urban vs. a rural area were 31% more likely to be non-persistent. The likelihood of non-persistence was highest in the polymedicated patients taking more than 10 concomitant drugs, while patients changing therapy (switching or addition) were more likely to persist with treatments.

**Table 4:** Predictors of non-persistence to antidiabetic therapy at 1-year post-initiation.

Characteristics	Unadjusted HR (95% CI)	*P*-value	Adjusted HR (95% CI)	*P*-value
**Age**				
40–59	0.977 (0.895–1.066)	0.601	1.094 (0.999–1.197)	0.052
60–79	Reference		Reference	
≥80	1.440 (1.265–1.639)	<0.001	1.268 (1.109–1.449)	0.001*
**Sex**				
Female	1.072 (0.988–1.162)	0.093	0.985 (0.906–1.071)	0.724
Male	Reference		Reference	
**Type of therapy in data index**				
Metformin	Reference		Reference	
Sulfonylureas	1.770 (1.572–1.993)	<0.001	1.697 (1.500–1.920)	<0.001*
**Area of living**				
Rural	Reference		Reference	
Urban	1.316 (1.124–1.540)	0.001	1.309 (1.117–1.533)	0.001*
**Polypharmacy**				
0–5 (no-polypharmacy)	Reference		Reference	
6–9 (polypharmacy)	1.122 (1.006–1.250)	0.038	1.131 (1.011–1.265)	0.032*
≥10 (Excessive polypharmacy)	1.512 (1.378–1.659)	<0.001	1.505 (1.359–1.668)	<0.001*
**Therapy change**				
No	Reference		Reference	
Yes	0.913 (0.816–1.020)	0.108	0.818 (0.728–0.919)	0.001*
**Dose change**				
None	Reference		Reference	
Decrease	0.552 (0.440–0.693)	<0.001	0.596 (0.474–0.750)	<0.001*
Increase	0.342 (0.282–0.415)	<0.001	0.355 (0.291–0.432)	<0.001*
**Insulin addition**				
No	Reference		Reference	
Yes	0.874 (0.683–1.119)	0.286	0.979 (0.760–1.262)	0.871
**Neuro-psychiatric drugs**				
No	Reference		Reference	
Yes	1.225 (1.111–1.352)	<0.001	1.058 (0.953–1.174)	0.292

*P-value less of 0.05 was considered to be statistically significant.

## Discussion

This study provides a comprehensive up-to-date overview of T2D treatment patterns among patients initiating antidiabetic therapies in a real-world context. Metformin was the most common initial treatment (80.3%), as recommended by international guidelines (American Diabetes Association, 2018a). By grouping patients in monotherapy with those in combination therapy, more than 90% of the study population was using metformin as initial therapy. The small percentage (7.7%) of patients who initiated with sulfonylurea in monotherapy may represent a diabetic population with metformin contraindication (Jermendy et al., 2012; Iolascon et al., 2016; Heintjes et al., 2017; American Diabetes Association, 2018a). It is disappointing to note that, despite current clinical guidelines where metformin is the first line of treatment (American Diabetes Association, 2018a), it was observed first-line prescription of sulfonylurea in 6.9% of patients aged 60–79 years and in 10.8% of patients aged 80 years and older. Repaglinide was also preferred in 9.1% of those aged ≥80 years. A recent study, exploring T2D treatment patterns across European countries, highlighted that repaglinide is often prescribed in Italy, more than in other countries (Heintjes et al., 2017). Furthermore, it is observed a very limited use of the recently introduced drugs such as SGLT2 inhibitors (empagliflozin) and GLP-1RAs (liraglutide), which have demonstrated a significant reduction in cardiovascular death (American Diabetes Association, 2018a). This less prescription may be due to the fact that our study population includes only new users of antidiabetics, and they are not used as first-line prescription (American Diabetes Association, 2018a).

Most of the patients starting with a combined treatment (74%) received a fixed-dose combination, which is only recommended in the guidelines as a first-line treatment with a high level of glycated haemoglobin values (American Diabetes Association, 2018a).

Our population was similar to the population diagnosed with diabetes, because the incidence of diabetes mellitus was higher in patients older than 60 years old, as it was observed in 2016 in Italy (Gargiulo et al., 2017). The new users of AD had a mean comorbidity score of 3.3 ± 2.7. It has been explained that diabetes is commonly associated with hypertension arterial, atherosclerotic cardiovascular disease (coronary heart disease, cerebrovascular disease, or peripheral arterial disease), and microvascular complications. Therefore, these diseases are contributor to the direct and indirect cost of diabetes, and controlling individual cardiovascular risk could prevent diabetes (Arrieta et al., 2015; American Diabetes Association, 2018b). Due to this relationship between diabetes and other cardiovascular diseases (Arrieta et al., 2015; American Diabetes Association, 2018b), 70% of the patients in treatment with metformin had an excessive polypharmacy. The patients were followed up over time and regimen changes occurred in about 22% of patients, with treatment switching (12.6%) being more frequent than treatment addition (9.1%).

Patients that started the treatment with metformin showed fewer treatment modifications compared to patients with sulfonylureas (10.4% vs 35.2%), similar to studies in The Netherlands (Lamberts et al., 2011) and Korea (Noh et al., 2018). An interesting finding is that 73.1% of patients initiated on a sulfonylurea received metformin as a regimen change: this trend was also observed in Irish cohort of newly diagnosed diabetes patients (Grimes et al., 2015). Metformin accounted also for 66.7% of treatment additions for those initiated on a sulphonylurea. High proportion of patients received a metformin prescription as a treatment addition. That treatment suggested that the initial choice of sulfonylurea was not due to a contraindication to metformin (Grimes et al., 2015).

A dose change occurred in 14.4% of the patients starting with metformin, compared to only 2.9% of patients starting with sulfonylurea. The observed difference could be due to the recommendation of gradual dose increase in the initial prescription of metformin to avoid gastrointestinal side effects, whereas the dose increase is not recommended in sulfonylureas for the risk of hypoglycaemic episodes at higher doses (Grimes et al., 2015).

Patients with metformin monotherapy as initial treatment often had an insulin treatment added to their treatment regimen. This could be due to a need for treatment intensification keeping metformin treatment in agreement with the diabetes guidelines (American Diabetes Association, 2018a). Second most frequent choice was a combination of sulfonylurea and DPP-4 inhibitors; other newer antidiabetic agents were prescribed much less frequently during treatment progression. A European cross-country comparison showed that, after metformin treatment, the most frequent combination was metformin and sulfonylurea in the Netherlands, United Kingdom, and Spain, while in Italy the use of multiple other treatments was observed (Overbeek et al., 2017).

Approximatively 20% of patients were non-persistent after 1 year of treatment in the current study. Similar results have been observed in Quebec, Canada, with 79.3% of the newly dispensed an ADs or insulin patients persistent after 1 year of treatment (Guénette et al., 2013) and 80.8% persistent after 2 years of the initiation of the AD treatment (Dossa et al., 2015). Therefore, in Ireland, Grimes et al. observed that 79% of patients on metformin were persistent 12 months after initiation, while 69% of patients were persistent with a sulphonylurea (Grimes et al., 2015). In Hungary, the 1-year rate of persistence with ADs proved to be surprisingly low, with 47.7% of patients persistent with metformin and 45.4% persistent with sulphonylurea treatment (Jermendy et al., 2012). In general, persistence to antidiabetic drugs ranged from 41.0% to 81.1% as shown in a meta-analysis of studies, published in 2015, that examined the adherence, persistence, and discontinuation for patients with an AD prescription (Iglay et al., 2015). In another study in Italy, the adherence to chronic medication was low and it was associated with the level of education (Menditto et al., 2015). The differences in the definition of persistence, the nature of the populations studied, and the time periods covered could explained this range of values in the persistence (Guénette et al., 2013).

Cox regression analysis showed that patients aged 80 years and older compared to younger populations patients who initiated sulfonylurea, experienced polypharmacy, and lived in an urban area were more likely to be non-persistent.

These factors should be taken into consideration by GPs and diabetologists when they initiate a hypoglycemic drug treatment in older people. Moreover, in patients with these factors, persistence to drug treatment should be monitored over time by the clinician.

However, there is no consensus on influence of these factors on persistence to treatment in the literature, in particular the influence of age. In some studies is shown an increase in adherence and persistence with age, and in others the opposite is observed (Pedan et al., 2007; Menditto et al., 2018; Moreno Juste et al., 2019). Usually, older age is associated with increased morbidity, frailty, and cognitive impairment, which can also increase the discontinuation of the treatment (Menditto et al., 2018).

Also, the effect of polypharmacy is inconclusive, with some studies showing a positive influence on persistence and others a negative influence (O’Shea and Teeling, 2013; Moreno Juste et al., 2019). To better assess the role of treatment complexity in antidiabetic treatment, more research on persistence is necessary in this area (Guénette et al., 2013).

Conversely, our finding of a relationship between urbanization and reduced persistence of AD treatment is mostly consistent with the literature. It has been reported in two studies in Quebec that patients living in rural areas were more likely to persist with their antidiabetic treatment compared with urban regions (Guénette et al., 2013; Simard et al., 2015). This may be related to a more active management of patients and better control of the treatment in rural areas (Scala et al., 2016). Therefore, urbanization is associated with an increased consumption of processed foods, lower physical activity, anxiety, and lack of sleep through residential noise, which are all risk factors for diabetes (DenBraver et al., 2018). In relation to treatment initiation, patients with metformin monotherapy were more likely to remain persistent when compared with sulfonylurea monotherapy, as observed in previous studies (Gregoire et al., 2010; Guénette et al., 2013; O’Shea and Teeling, 2013; Grimes et al., 2015; Simard et al., 2015). AD-related side effects, such as hypoglycemic events, have been suggested to be a significant barrier to persistence (Guénette et al., 2013). On the other hand, patients who changed therapy and experienced dose changes were more likely to be persistent.

## Strengths and Limitations

This is the first Italian study investigating T2D treatment patterns and including new drug classes such as GLP-1Ras or SGLT2 inhibitors. This study adds to the frame of existing knowledge on prescription profiles of T2D drugs. Our study is based on a data source with full coverage of T2D prescriptions for a stable population and a region defined. In addition, this database has multiple variables such as age, gender, co-morbidity, and co-medications. With this analysis, characterization of the use of antidiabetic therapy in a regional context is explored and it is useful for exploring the dynamics of the diabetes treatment.

However, this study also has potential limitations. Firstly, the study does not cover the entire Italian population, but in Italy, there is a uniform health service in all different regions and it is plausible that prescription patterns in this region are similar to the rest of Italy. The use of administrative databases does not allow the detection of clinical information such as changes in lifestyle (e.g., better diet quality and weight loss), glycated haemoglobin values, and medical reasons for treatment discontinuation. Also, the changes in the drug usage (e.g., pillbox use) are not documented in administrative databases. Finally, the medication prescribed does not ensure that the medication was taken. Nonetheless, the measure of medication persistence used in this study has been validated for use in others studies (Simard et al., 2015; Menditto et al., 2017). Persistence may have been overestimated in cases where individuals filled their prescriptions but did not take the drug, because this database is based on patterns of drugs dispensed, but not necessarily consumed.

Finally, it has been reported in the literature that around 60% of patients who discontinue their AD treatment initiate a new course of treatment within the year following discontinuation (Guénette et al., 2013). For this reason, it cannot be assumed that patients who had not filled a prescription for an antidiabetic treatment in the following year to the data index will never again take any such treatment, so these patients cannot be classified as non-persistents.

The generalizability of our results is restricted as the healthcare systems, reimbursement policies, and access to different treatment options are country-specific. In this regard, further research should focus on cross-country comparison.

## Conclusions

Our study shows that treatment of T2D in Italy is in general consistent with clinical guidelines (in particular, in relation to the large use of metformin as first step therapy and the more prominent use of newer antidiabetic agents during disease progression). However, the last seem not to be preferred in patients with a higher comorbidity score, although these patients could benefit from this kind of treatment. In addition, it was observed that patients starting treatment with metformin showed fewer treatment modifications compared to patients initiating with sulfonylureas. Persistence to treatment was relatively high in our study in comparison to others previously published. Persistence with treatment was lower in those receiving sulphonylureas, living in an urban area, and with higher polypharmacy. These findings still deserve attention and should be addressed in future treatment guidelines.

Our findings in patients’ characteristics might help identify those patients who would benefit from counseling from their health care practitioner on better antidiabetic drugs usage. Research is needed to increase long-term persistence and to improve antidiabetic drugs use and glycemic control in T2D especially among newly diagnosed patients. Providing information based on real-world data may be a useful way to explore the dynamics of antidiabetic therapy within a specific context and to optimize the use of resources for a better management of the disease.

## Contribution to the Field Statement

Type 2 diabetes (T2D) is widely considered one of the world’s largest human health problems, as documented by its growing prevalence in recent decade. In 2016, more than 3.2 million people in Italy reported to suffer from diabetes, 5.3% of the total population. Over the last decade, several new antidiabetic drugs (ADs), such as DPP-4, SGLT2 inhibitors, and GLP-1Ras, with varying clinical efficacy, profiles, and costs, are being introduced in the market, enabling physicians to tailor therapy for each individual patient. The drug-utilization study, proposed here, describes up-to-date pattern of utilization of ADs in a real-world context exploring therapy switching, add-on, and persistence to therapy in a large population of new users of antidiabetic drugs. Newer antidiabetic agents are used during disease progression. However, they seem not to be preferred in patients with a higher comorbidity score, although these patients could benefit from this kind of treatment. Factors related to therapy discontinuation were also investigated. Persistence with treatment was lower in those receiving sulphonylureas, living in an urban area, and with higher polypharmacy. These findings still deserve attention and should be addressed in future treatment guidelines.

## Data Availability

The datasets for this manuscript are not publicly available because the data set was only accessed and analyzed by the authors who are affiliates to CIRFF, University of Naples. Authors who are not affiliates received the results from the analysis of the data for discussion. Access to the data is allowed only to affialiates due to Campania region policies. Requests to access the datasets should be directed to enrica.menditto@unina.it.

## Author Contributions

AMJ and EM conceived and designed the study. VMM, VO, and AMJ performed the analysis. All authors have contributed to the review strategy and interpretation of the results. APT and GR supervised the study. AMJ drafted the initial version of the manuscript. EM, VO, CC, APT, AGM, FGR, MAP, and GR contributed to refining and critically reviewed the manuscript for intellectual content. The CIRFF coordinated the study analysis. All authors approved the final manuscript as submitted.

## Funding

AJ received a Grant for Resident Researchers from Fundación Instituto de Investigación Sanitaria Aragón (IIS Aragón).

## Conflict of Interest Statement

The authors declare that the research was conducted in the absence of any commercial or financial relationships that could be construed as a potential conflict of interest.
