# Supplementary material for: Treatment Patterns of Diabetes in Italy: A Population-Based Study
Source: Front Pharmacol. 2019 Aug 6;10:870. doi: 10.3389/fphar.2019.00870 (PMC6691351; doi:10.3389/fphar.2019.00870)
Supplement: Supplementary file 1 [file Table_1.docx]

**Supplementary Table 1. Rx-risk comorbidity category and corresponding ºATC codes**

| **Rx-risk comorbidity category** | **ATC code** |
| --- | --- |
| Alcohol dependency | N07BB01–N07BB99 |
| Allergies | R01AC01–R01AD60, R06AD02–R06AX27, R06AB04 |
| Anticoagulants | B01AA03–B01AB06, B01AE07, B01AF01, B01AF02, B01AX05 |
| Antiplatelets | B01AC04–B01AC30 |
| Anxiety | N05BA01–N05BA12, N05BE01 |
| Arrhythmia | C01AA05, C01BA01–C01BD01, C07AA07 |
| Benign prostatic hyperplasia | G04CA01–G04CA99, G04CB01, G04CB02* |
| Bipolar disorder | N05AN01 |
| Chronic airways disease | R03AC02–R03DC03, R03DX05 |
| Congestive heart failure | C03DA02–C03DA99, C07AB02, C07AB07, C07AG02, C07AB12, C03DA04 (C03CA01– C03CC01 and C09AA01–C09AX99, C09CA01– C09CX99)† |
| Dementia | N06DA02–N06DA04, N06DX01 |
| Depression | N06AA01–N06AG02, N06AX03–N06AX11, N06AX13–N06AX18, N06AX21–N06AX26 |
| Epilepsy | N03AA01–N03AX99 |
| Glaucoma | S01EA01–S01EB03, S01EC03–S01EX99 |
| Gastrooesophageal reflux disease | A02BA01–A02BX05 |
| Gout | M04AA01–M04AC01 |
| Hepatitis B | J05AF08, J05AF10, J05AF11 |
| Hepatitis C | J05AB54, L03AB10, L03AB11, L03AB60, L03AB61, J05AE14, J05AE11–J05AE12, J05AX14, J05AX15, J05AX65, J05AB04 |
| HIV | J05AE01–J05AE10, J05AF12–J05AG05, J05AR01–J05AR99, J05AX07–J05AX09, J05AX12, J05AF01–J05AF07, J05AF09 |
| Hyperkalaemia | V03AE01 |
| Hyperlipidaemia | C10AA01–C10BX09 |
| Hypertension | C03AA01–C03BA11, C03DB01, C03DB99, C03EA01, C09BA02–C09BA09, C09DA02– C09DA08, C02AB01–C02AC05, C02DB02– C02DB99 (C03CA01–C03CCO1 or C09CA01– C09CX99)§ |
| Hyperthiroidism | H03BA02, H03BB01 |
| Hypothiroidism | H03AA01–H03AA02 |
| Irritable bowel syndrome | A07EC01–A07EC04, A07EA01–A07EA02, A07EA06, L04AA33 |
| Ischaemic heart disease: angina | C01DA02–C01DA14, C01DX16, C08EX02 |
| Ischaemic heart disease: hypertension | C07AA01–C07AA06, C07AA08–C07AB01, C07AB02, C07AG01, C08CA01–C08DB01, C09DB01–C09DB04, C09DX01, C09BB02– C09BB10, C07AB03, C09DX03, C10BX03¶ |
| Incontinence | G04BD01–G04BD99 |
| Inflammation/pain | M01AB01–M01AH06 |
| Liver failure | A06AD11, A07AA11 |
| Malignancies | L01AA01–L01XX41 |
| Malnutrition | B05BA01–B05BA10 |
| Migraine | N02CA01–N02CX01 |
| Osteoporosis/Paget's | M05BA01–M05BB05, M05BX03, M05BX04, G03XC01, H05AA02 |
| Pain | N02AA01–N02AX02, N02AX06, N02AX52, N02BE51 |
| Pancreatic insufficiency | A09AA02 |
| Parkinson's disease | N04AA01–N04BX02 |
| Psoriasis | D05AA01–D05AA99, D05BB01 D05BB02, D05AX02, D05AC01–D05AC51, D05AX52 |
| Psychotic illness | N05AA01–N05AB02, N05AB06–N05AL07, N05AX07–N05AX13 |
| Pulmonary hypertension | C02KX01–C02KX05 |
| Renal disease | B03XA01–B03XA03, A11CC01–A11CC04, V03AE02, V03AE03, V03AE05 |
| Smoking cessation | N07BA01–N07BA03, N06AX12 |
| Steroid-responsive disease | H02AB01–H02AB10 |
| Transplant | L04AA06, L04AA10, L04AA18, L04AD01, L04AD02 |
| Tubercolosis | J04AC01–J04AC51, J04AM01–J04AM99 |

ºATC code: Anatomical Therapeutic Chemical code

†Must have at least two medicines prescribed with one of those medicines having an ATC code from C03CA01–C03CC01 and the other having an ATC code from either C09AA01–C09AX99 or C09CA01–C09CX99.

§Can have medicine dispensed with an ATC code C03CA01–C03CC01 or C09AA01–C09AX99, but not both, as this would indicate chronic heart failure.

¶Combination product for hyperlipidaemia and ischaemic heart disease: hypertension. N/A, not applicable.

| **Supplementary Table 2. Characteristics of subjects receiving only one prescription of antidiabetic drugs in the observation period (spot therapy).** | | | | | | | | | |
| --- | --- | --- | --- | --- | --- | --- | --- | --- | --- |
| **Characteristics** | |  |  | **Monotherapy N=3825 (78.6%)** | |  |  | **Combination Therapy N=1042 (21.4%)** | |
|  | **Total N=4867** | **Metformin N=2585 (67.6%)** | **Sulfonylureas N=559 (14.6%)** | **Alpha glucosidase inhibitors N=194 (5.1%)** | **Repaglinide N= 268 (7.0%)** | **£DPP-4 inhibitor N=153 (4.0%)** | **Other monotherapy**  **N= 66 (1.7%)** | **Fixed Combination N=929 (89.1%)** | **Free Combination N=113 (10.8%)** |
|  |  |  |  |  |  |  |  |  |  |
|  |  |  |  |  |  |  |  |  |  |
| **Age (MD±SD)** | 61.6±19.4 | 61.3±17.8 | 63.7±19.0 | 65.7±17.9 | 68.9 ± 16.7 | 58.9±26.4 | 57.9±20.8 | 59.1±22.3 | 60.7±20.1 |
| **Age** |  |  |  |  |  |  |  |  |  |
| **40-59** | 1332 (27.3%) | 773 (29.8%) | 141 (25.2%) | 40 (20.6%) | 53 (19.7%) | 29 (19.0%.) | 15 (22.7%) | 246 (26.4%) | 35 (30.7%) |
| **60-79** | 2136 (43.7%) | 1159 (44.7%) | 248 (44.3%) | 94 (48.5%) | 124 (46.1%) | 57 (37.3%) | 34 (51.5%) | 366 (39.2%) | 54 (47.4%) |
| **≥80** | 803 (16.4%) | 358 (13.8%) | 114 (20.4%) | 41 (21.1%) | 77 (28.6%) | 33 (21.6%) | 6 (9.1%) | 161 (17.3%) | 13 (11.4%) |
| **Sex** |  |  |  |  |  |  |  |  |  |
| **F** | 2501 (51.2%) | 1362(52.7%) | 280 (50.1%) | 114 (58.8%) | 125 (46.6%) | 83 (54.2%) | 35 (53.0%) | 452 (48.7%) | 50 (44.2%) |
| **M** | 2366 (48.4%) | 1223 (47.3%) | 279 (49.9%) | 80 (41.2%) | 143 (53.4%) | 70 (54.1%) | 31 (47.0%) | 477 (51.3%) | 63 (55.8%) |

£ DPP-4: dipeptidyl peptidase-4 inhibitors

| **Supplementary Table 3. Distribution of fixed combinations at treatment initiation by ºATC code** | |
| --- | --- |
| **ATC** | **N (%)** |
| **A10BD02** (Metformin and sulfonylureas) | 756 (53%) |
| **A10BD05** (Metformin and pioglitazone) | 72 (5%) |
| **A10BD06** (Glimepiride and pioglitazone) | 6 (0.4%) |
| **A10BD07** (Metformin and sitagliptin) | 180 (12.6%) |
| **A10BD08** (Metformin and vildagliptin) | 148 (10.4%) |
| **A10BD09** (Pioglitazone and alogliptin) | 10 (0.7%) |
| **A10BD10** (Metformin and saxagliptin) | 13 (0.9%) |
| **A10BD11** (Metformin and linagliptin) | 111 (7.8%) |
| **A10BD13** (Metformin and alogliptin) | 49 (3.4%) |
| **A10BD15** (Metformin and dapagliflozin) | 36 (2.5%) |
| **A10BD16** (Metformin and canagliflozin) | 21 (1.5%) |
| **A10BD20** (Metformin and empagliflozin) | 24 (1.7%) |
| **Total** | 1426 (9.7%) |

ºATC code: Anatomical Therapeutic Chemical code
